# Current Trends in Recombinant Human Bone Morphogenetic Protein 2 (rhBMP2) Usage for Spinal Fusion Surgery

**DOI:** 10.3390/medicina59050878

**Published:** 2023-05-03

**Authors:** Harshadkumar A. Patel, Ian J. Wellington, Klair Lubonja, John W. Stelzer, Christopher L. Antonacci, Ergin Coskun, Mark P. Cote, Hardeep Singh, Scott S. Mallozzi, Isaac L. Moss

**Affiliations:** 1 Westchester Medical Center, Department of Orthopaedic Surgery, Valhalla, NY 10595, USA; 2 Department of Orthopaedic Surgery, University of Connecticut, Farmington, CT 06108, USA; 3 Department of Orthopedic Surgery, Massachusetts General Hospital, Boston, MA 02114, USA

**Keywords:** fusion, bone morphogenetic protein, BMP, rhBMP2, augmentation, on-label, off-label

## Abstract

(1) *Background*: Since first approved by the FDA, on-label and off-label usage of recombinant human bone morphogenetic protein 2 (rhBMP2) for spinal fusion surgeries has become widespread. While many studies have investigated the safety and efficacy of its use, as well as its economic impact, few have looked at the current trends in its on- and off-label use. The goal of this study is to evaluate the current trends of on- and off-label rhBMP2 use for spinal fusion surgery. (2) *Methods*: A deidentified survey was created and electronically distributed to members of two international spine societies. Surgeons were asked to report their demographic information, surgical experience, and current usage of rhBMP2. They were then presented with five spinal fusion procedures and asked to report if they use rhBMP2 for these indications in their current practice. Responses were stratified between rhBMP2 users vs. non-users and on-label vs. off-label use. Data were analyzed using chi-square with Fisher’s exact test for categorical data. (3) *Results*: A total of 146 respondents completed the survey with a response rate of 20.5%. There was no difference in overall rhBMP2 usage based on specialty, experience, or number of cases per year. Fellowship-trained surgeons and those who practice in the United States were more likely to use rhBMP2. Surgeons who were trained in the Southeast and Midwest regions reported the highest usage rates. rhBMP2 use was more common among fellowship-trained and US surgeons for ALIFs; non-US surgeons for multilevel anterior cervical discectomy and fusions; and fellowship-trained and orthopedic spine surgeons for lateral lumbar interbody fusions. Non-US surgeons were more likely to use rhBMP2 for off-label indications compared to surgeons from the US. (4) *Conclusions*: While various demographics of surgeons report different rates of rhBMP2 use, off-label use remains relatively commonplace amongst practicing spine surgeons.

## 1. Introduction

Operative treatment of spine pathology often involves the fusion of vertebral bodies. This is often carried out to provide stability, prevent or correct deformity, and/or to decrease the chance of developing future pathologies. While the fusion of vertebral bodies is a common goal in spine surgery, it is not a certain outcome. Failure of fusion between vertebral bodies can result in the development of a symptomatic pseudoarthrosis which is a painful nonunion between two boney segments. Pseudoarthrosis can result in significant patient morbidity and is responsible for increased healthcare costs and utilization [1]. Given the negative effects that a symptomatic pseudarthrosis can exert on both a patient and on the healthcare system in general, treating surgeons are encouraged to decrease the risk of a patient developing a pseudoarthrosis to the best of their ability. Grafting has become a common method to increase a patient’s fusion potential. Currently, many graft options, both autograft and allograft, are available to surgeons, with surgeon preference often determining which graft is utilized.

Bone-morphogenetic proteins (BMPs) are a family of osteoinductive growth factors which are intimately involved with the process of bone formation [2]. Human recombinant BMP-2 (rhBMP2) functions by inducing the expression of osteocalcin in osteoblasts [3]. In 2002, rhBMP2, marketed as Infuse (Medtronic, Minneapolis, MN), became FDA-approved for fusion augmentation of single-level anterior lumbar interbody fusions (ALIF) [4]. Studies on rhBMP-2 have demonstrated decreased operating times, improvement in clinical outcomes, and shorter hospital stays when compared with autografts alone [5]. Given its success, surgeons have begun to use rhBMP2 “off-label” in various spinal fusion surgeries to enhance fusion potential, with the aims of preventing the development of pseudoarthrosis.

A 2010 study of the National Inpatient Sample (NIS) database showed that nearly 85% of all the spine surgeries in which BMP was used were for “off-label” indications [6]. While studies did show that rhBMP2 was successful at augmenting spinal fusions, they also discovered complications associated with its use. For example, cases of paravertebral swelling leading to airway obstruction have been reported with its use in anterior cervical discectomy and fusions (ACDFs) [7]. Additionally, studies have found a possible increase in risk of malignancy associated with its use [8]. Finally, non-life-threatening complications such as dysphagia, local inflammation, sterile cyst formation, osteolysis, implant migration, neuritis, and ectopic bone formation have also been observed [9,10,11]. Khan et al. in 2017 found that the off-label use of rhBMP-2 was effective for achieving bone fusion with lower rates of non-union, but higher rates of associated radiculitis and seroma [12]. Hofstetter et al. found that the frequency of complications from rhBMP-2 rose with increasing dosages used [13]. Importantly, the rate of rhBMP-2 use in the United States has been reported to be declining steadily since 2008, possibly due to the reports of possible associated adverse effects [14].

Given that the “off-label” use of rhBMP2 has been shown to successfully increase fusion rates but is associated with various complications as well as increased healthcare expenditure [15], it is important to understand how frequently it is being used in this fashion and in what situations it is being utilized. There have been many studies to date investigating trends in general rhBMP2 usage and the rates of usage for different patient populations [10,16,17,18]. To date, there have been no published studies investigating the current trend of on- and off-label rhBMP2 use among spinal surgeons. This study aims to identify current trends in on- and off-label rhBMP-2 use in spinal fusion surgery among global spine surgeons.

## 2. Materials and Methods

This study was approved by our institutional review board prior to initiation (#21x-161-2). A survey was created using Research Electronic Data Capture (REDCap^TM^) data collection software. Participants provided informed consent prior to initiation of the survey. The survey contained three parts. The first section asked for demographic information and surgical experience. The second section inquired about each respondent’s current usage of rhBMP-2 for lumbar and cervical spine fusion surgery. The third section asked each respondent about their usage of rhBMP-2 in five different spinal fusion surgeries: single level ALIF, multilevel ACDF, single level open transforaminal lumbar interbody fusion (TLIF), minimally invasive (MIS) TLIF, and lateral lumbar interbody fusion (LLIF). The survey was distributed electronically to the members of two independent spine surgery societies: the International Society for the Advancement of Spine Surgery (ISASS) and the Society of Minimally Invasive Spine Surgery (SMISS). Two reminder emails were sent at two-week intervals. The survey was left open for a total of 6 weeks. Incomplete surveys were not included in the analysis.

### Statistical Analysis

Respondents were classified as rhBMP2 users and non-users. Users’ responses were further evaluated for rate of on-label use (those who use rhBMP2 only for ALIF surgery) and off-label use (those who use rhBMP2 for non-FDA-approved spine fusion surgeries). Chi-square with Fisher’s exact testing was used to compare categorical data based on different respondent demographics. To account for regional differences in practice patterns, the proportion of rhBMP2 use was post-stratified based on region of practice. Sampling weights by US region of practice were derived from the American Academy of Orthopedic Surgeons (AAOS) 2018 census [19]. Data for each state were combined into five distinct regions: Northeast, Southeast, Midwest, Southwest, and West. Sampling weights were then used to adjust survey responses to BMP use according to US region of practice. All analyses were performed with Stata Statistical software (StataCorp. 2017. Stat Statistical Software: Release 15. College Station, TX, USA: StataCorp LLC).

## 3. Results

### 3.1. Respondents

Surveys were distributed to 712 surgeons and were completed by 146 respondents, yielding a response rate of 20.5%.

### 3.2. Overall rhBMP2 Use

Usage of rhBMP2 augmentation in any capacity was reported by 102 respondents, while 40 denied any usage of rhBMP2. There was no difference in overall usage based on specialty (*p* = 0.95), experience (*p* = 0.06), practice setting (*p* = 0.861), number of spine surgery cases per year (*p* = 0.86), or the percentage of spine surgery of overall surgical practice (*p* = 0.07) (Table 1). Those who were fellowship-trained were significantly more likely to report rhBMP2 use than those who were not fellowship-trained (*p* = 0.009). Additionally, those practicing in the United States (US) were more likely to report rhBMP2 use than their international counterparts (*p* = 0.001). A total of 30.39% of respondents who use rhBMP2 augmentation reported a decrease in usage over the past 5 years, 21.57% reported an increase over the same time period, while 48.04% reported no change in their frequency of use.

### 3.3. Use of rhBMP2 by Anatomic Location

There was no difference in the rate of rhBMP2 usage for lumbar fusion surgery based on surgeon experience (*p* = 0.73), specialty (*p* = 0.36), fellowship training (*p* = 0.36), practice setting (*p* = 0.308), practice within the US (*p* = 0.36), or region of practice within the US (*p* = 0.11) (Table 2). There was a difference in rate of rhBMP2 usage based on region of fellowship training within the US (*p* = 0.016), with those who underwent fellowship training in the Southeast and Midwest reporting the highest rates of usage (Table 2). There was no difference in the rate of rhBMP2 usage in cervical fusion cases based on experience, specialty, fellowship training, practice setting, region of fellowship training, practice within the US, or region of practice with the US (Table 2).

### 3.4. Use of rhBMP2 by Procedure

Use of rhBMP2 for ALIF procedures was reported more often in surgeons who were fellowship-trained (*p* = 0.002) and those who practice in the US (*p* = 0.027). There was no difference in the rate of use for ALIF procedures based on experience, specialty, practice setting, region of fellowship training, or region of practice within the US (Table 3).

rhBMP2 use in multilevel ACDFs was more common in those practicing outside of the US (*p* = 0.027). The remaining demographics had no effect on rates of use in multilevel ACDF (Table 3).

The only factor affecting rates of rhBMP2 use in open TLIF surgery was region of fellowship training (*p* = 0.04), with surgeons who underwent fellowship training in the Midwest reporting the highest rates of use (Table 3). No respondent who underwent fellowship training in the West reported using rhBMP2 for open TLIF augmentation.

There was no effect of any of the demographics evaluated on the rates of rhBMP2 use for MIS TLIF surgery (Table 3). However, for LLIF procedures, rhBMP2 was used more often by orthopedic spine surgeons (*p* = 0.042), in those with fellowship training (*p* = 0.027), and in those practicing in the Southwest or Midwest (*p* = 0.012) (Table 3).

### 3.5. On-Label and Off-Label Use of rhBMP2

Overall, 89% of respondents who reported using rhBMP2 stated they used it off-label. The rate of off-label compared to on-label use of rhBMP2 was more commonly reported in those practicing outside of the US (100%) compared to those practicing within the US (84.9%) (*p* = 0.043). There was no effect of surgical experience, specialty, fellowship training, practice setting, region of fellowship training, or region of practice within the US on rates of overall off-label use (Table 4).

### 3.6. Size of rhBMP2 Used by Type of Surgery

For those that endorsed usage of rhBMP2, the size of rhBMP2 used for various procedures was determined and is reported in Table 5. The small size was most commonly used in ALIF, open TLIF, MIS TLIF, and LLIF procedures. The XX small size was the most commonly reported size for multilevel ACDFs (Table 5).

### 3.7. Use of rhBMP2 by Region of Practice

Among 146 respondents, 100 were currently practicing in the US. Among these 100, 33 (33%) were in the Southeast, 20 were in the Northeast (20%), 20 were in the West (20%), 18 were in the Midwest (18%), and 9 (9%) were in the Southwest. This distribution was consistent with the 2018 AAOS census (Table 6). When use of rhBMP2 was stratified by region, there was minimal effect on overall rhBMP2 use or use in any of the five evaluated procedures (Table 7).

## 4. Discussion

Our study successfully identified current trends in on- and off-label use of rhBMP2 in spinal fusion surgery. We found that, compared to surgeons outside of the US, surgeons in the US showed higher rates of BMP usage overall, as well as higher rates of usage in ALIFs (on-label). Surgeons outside of the United States reported higher rates of off-label usage in general and a higher rate of usage in multilevel ACDFs. This trend of higher off-label usage outside of the United States may be due to different legal environments surrounding off-label use of products in other countries. Fellowship training was associated with higher overall usage, as well as off-label usage in LLIFs. Additionally, orthopedic spine surgeons reported higher rates of rhBMP2 usage in LLIFs compared to neurosurgeons. Finally, our study demonstrated that region of practice and region of fellowship training had an effect on the rates of rhBMP2 usage for various procedures. Region, fellowship, and specialty differences of rhBMP2 are somewhat intuitive. Given that off-label use is based on surgeon preference rather than established practice guidelines, surgeons who were trained by someone using rhBMP2 in an off-label fashion may be more inclined to use it in their practice. Additionally, surgeons in a particular region where off-label use is more common may be more inclined to use it as well to match their peers. The flexible indications for off-label rhBMP2 use would inherently result in pockets of surgeons with similar training backgrounds and practice settings who are more likely to use rhBMP2.

Our study demonstrated that 89% of surgeons reported the use of rhBMP2 off-label. Ong et al. in 2010 found that 85% of the spinal fusion surgeries utilizing rhBMP2 were for an off-label application [6]. There has been an increasing trend of rhBMP2 use in ACDF surgery from 2% in 2002 to 10.7% in 2007. However, more recent studies have identified a decreasing trend in the use of off-label rhBMP2 usage. Poeran et al. found a decrease in the off-label use of rhBMP2 in ACDFs from 5.1% to 2% from 2006 to 2012, respectively [20]. They also described a decrease in the usage for posterior cervical fusion from 15.3% to 8.5% in these same years. Our survey showed that only 9.28% of US surgeons use rhBMP2 for multilevel ACDF, resembling the decreasing trend of usage for cervical fusion surgery.

The use of rhBMP2 to augment spinal fusion has been extensively evaluated. For on-label use, Burkus et al. found that the use of rhBMP2 use in ALIF surgery resulted in decreased pain and improved outcomes compared with traditional iliac crest bone grafting [21]. Additionally, they found fusion rate of 100% with use of rhBMP2. Off-label, Glassman et al. evaluated 1037 patients who underwent posterolateral fusion with rhBMP2 augmentation and found that only 0.6% of patients experienced a complication which could be attributed to the rhBMP2 augmentation [22]. Additionally, a study in 2008 showed that while the initial cost of surgery utilizing rhBMP2 augmentation was higher compared to surgeries using iliac crest bone graft (USD 24,736 vs. USD 21,138) the use of rhBMP2 was associated with lower overall physician costs, lower postoperative rehab costs, and lower total combined costs at 3 months (USD 33,860 vs. USD 37,227) [23].

While the potential benefits of rhBMP2 use to augment fusion have been described, associated risks have been similarly shown. Shields et al. in 2006 described complications following ACDF with off-label rhBMP2 augmentation in 23.2 percent of patients [7]. A similar study identified an increase in postoperative swelling in patients undergoing ACDF with rhBMP2 [24]. In posterior cervical arthrodesis, rhBMP2 use has been associated with an increased risk of wound complications when compared to iliac crest bone grafting [25]. The increased bone formation associated with rhBMP2 use may cause adverse events itself. Chen et al. in 2010 described four cases of symptomatic ectopic bone formation following rhBMP2 augmentation for TLIF procedures [26]. Wong et al. in 2008 described five cases of neurologic deficits attributed due to ectopic bone formation following rhBMP2 use in PLIF and TLIF procedures [27]. Our study showed that even though overall BMP usage is greater among US surgeons compared to non-US surgeons (*p* = 0.001), US-based surgeons were less likely to use it in the cervical spine (*p* = 0.027). This suggests that US surgeons are expanding the usage of rhBMP2 to improve fusion, but at the same time they are cautious regarding its potential for side effects when used for certain indications.

Our study found that the region of fellowship training has a significant impact on the rhBMP2 usage practice among US surgeons. In general, surgeons are more prone to adopt new techniques or procedures taught during their fellowship. In the light of safety and efficacy literature for rhBMP2, it is the professional obligation for fellowship training centers to teach the judicial and appropriate use of rhBMP2 in spine fusion surgery.

While our study successfully identified the current usage of rhBMP2 for spinal fusion both on- and off-label, it was not without limitations. This was a survey study, and as such is subject to response bias. Respondents may have been more likely to participate due to being advocates for rhBMP2 usage, or they may be individuals who are staunchly against the off-label use. We did observe a response rate of 20.5%, which is similar to prior studies [14]. Furthermore, the relatively low number of respondents is a limitation of this study. Implementation of a survey incentive may have been an effective way of increasing the response rate of this study; however, this introduces other forms of bias. Additionally, participants in our study may have been wary of truthfully detailing their rate of off-label rhBMP2 usage, or they may not accurately estimate their actual usage. Finally, our study grouped participants into practicing within the United States and practicing outside of the United States, and did not further subclassify the countries of practice of those practicing outside of the United States. Further study is warranted to better understand the global rates of on- and off-label use of rhBMP2.

## 5. Conclusions

Our study was able to identify the current rates of on- and off-label usage of rhBMP2 among global spine surgeons. While different demographics of surgeons report different rates of use, off-label use remains relatively commonplace amongst practicing spine surgeons.

## Figures and Tables

**Table 1:** Overall BMP use among surgeons.

		Overall BMP Use		
		Yes	No	*p* Value
Respondents		102	40	
Specialty	Orthopedics	72 (72.00%)	28 (28.00%)	0.945
	Neurosurgery	30 (71.43%)	12 (28.57%)	
Fellowship training	Yes	88 (77.19%)	26 (22.81%)	0.009
	No	14 (50%)	14 (50%)	
Practice setting	Academic	22 (75.65%)	7 (24.14%)	0.861
	Private	60 (70.59%)	25 (29.41%)	
	Both	20 (71.43%)	8 (28.57%)	
Region of practice	US	78 (80.41%)	19 (19.59%)	0.001
	Non-US	23 (53.49%)	20 (46.51%)	
Experience	<10	13 (86.67%)	2 (13.33%)	0.060
	>10	32 (61.54%)	20 (38.46%)	
Spine surgery as a percentage of overall surgical practice	<50%	4 (44.44%)	5 (55.56%)	0.071
	>50%	98 (73.68%)	35 (26.32%)	
Spine surgery cases/year	<100	17 (70.83%)	7 (29.17%)	0.856
	>100	85 (72.65%)	32 (27.35%)	

**Table 2:** Demographic differences according to BMP use for lumbar and cervical fusion surgeries.

		Lumbar Fusion BMP Use		*p* Value	Cervical Fusion BMP Use			*p* Value
		Low	High		Don’t Use	Low	High	
Experience	<10	7 (53.85%)	6 (46.15%)	0.734	9 (69.23%)	4 (30.77%)	0	0.154
	>10	19 (59.38%)	13 (40.63%)		13 (40.63%)	15 (46.88%)	4 (12.5%)	
Specialty	Orthopedics	36 (50%)	36 (50%)	0.357	44 (61.11%)	23 (31.94%)	5 (6.94%)	0.077
	Neurosurgery	18 (60%)	12 (40%)		11 (36.67%)	16 (53.33%)	3 (10%)	
Fellowship training	Yes	45 (51.14%)	43 (48.85%)	0.360	47 (53.41%)	35 (39.77%)	6 (6.82%)	0.526
	No	9 (64.29%)	5 (35.71%)		8 (57.14%)	4 (28.57%)	2 (14.29%)	
Practice setting	Academic	14 (63.64%)	8 (36.36%)	0.308	10 (45.45%)	12 (54.55%)	0 (0%)	0.096
	Private	12 (60%)	8 (40%)		8 (40%)	9 (45%)	3 (15%)	
	Both	28 (46.67%)	32 (53.33%)		37 (61.67%)	18 (30%)	5 (8.33%)	
Region of fellowship in USA	Northeast	10 (50%)	10 (50%)	0.016	11 (55%)	6 (30%)	3 (15%)	0.19
	Southeast	2 (25%)	6 (75%)		2 (25%)	6 (75%)	0	
	Midwest	8 (30.77%)	18 (69.23%)		14 (53.85%)	12 (46.15%)	0	
	West	10 (76.92%)	3 (23.08%)		9 (69.23%)	4 (30.77%)	0	
	Southwest	5 (83.33%)	1 (16.67%)		4 (66.67%)	2 (33.33%)	0	
Practice within the US	US	39 (50%)	39 (50%)	0.359	41 (52.56%)	33 (42.30%)	4 (5.13%)	0.099
	Non-US	14 (60.87%)	9 (39.13%)		12 (54.55%)	6 (27.28%)	4 (14.29%)	
Region of practice in US	Northeast	11 (64.71%)	6 (35.29%)	0.116	13 (76.47%)	4 (23.53%)	0	0.171
	Southeast	12 (41.38%)	17 (58.62%)		14 (48.28%)	12 (41.38%)	3 (10.34%)	
	Midwest	3 (25%)	9 (75%)		4 (33.33%)	8 (66.67%)	0	
	West	9 (69.23%)	4 (30.77%)		6 (46.15%)	7 (53.85%)	0	
	Southwest	4 (57.14%)	3 (42.86%)		4 (57.14%)	2 (28.57%)	1 (14.29%)	

**Table 3:** BMP use trends for various fusion surgeries.

		ALIF	*p*-Value	Multilevel ACDF	*p*-Value	Open TLIF	*p*-Value	MIS TLIF	*p*-Value	LLIF	*p*-Value
BMP Use		81 (66.39%)		18 (13.04%)		61 (46.92%)		59 (46.46%)		62 (57.41%)	
Experience	<10	12 (80%)	0.122	0	0.105	7 (58.33%)	0.438	6 (50%)	0.708	8 (53.33%)	0.52
	>10	26 (57.78%)		8 (15.38%)		22 (45.83%)		22 (44%)		17 (43.59%)	
Specialty	Orthopedics	62 (70.45%)	0.1	12 (12.9%)	0.906	39 (43.33%)	0.272	35 (41.67%)	0.164	47 (63.51%)	0.042
	Neurosurgery	18 (54.55%)		6 (13.64%)		21 (53.85%)		23 (54.76%)		14 (42.42%)	
Fellowship training	Yes	71 (72.45%)	0.002	15 (13.89%)	0.616	52 (49.52%)	0.151	49 (49%)	0.19	54 (62.07%)	0.027
	No	9 (39.13%)		3 (10.33%)		8 (33.33%)		9 (34.62%)		7 (35%)	
Practice setting	Academic	17 (70.83%)	0.891	1 (3.57%)	0.102	13 (50%)	0.053	11 (45.83%)	0.821	13 (52%)	0.724
	Private	49 (65.33%)		11 (13.10%)		31 (39.24%)		13 (52%)		61 (57.01%)	
	Both	14(63.64%)		6 (24%)		16 (66.67%)		34 (44.16%)		34 (56.67%)	
Region of fellowship in USA	Northeast	17 (80.92%)	0.176	5 (20.83%)	0.087	12 (50%)	0.04	13 (59.09%)	0.146	15 (78.95%)	0.077
	Southeast	9 (90%)		2 (20%)		6 (60%)		6 (60%)		5 (62.5%)	
	Mid-west	21 (84%)		1 (3.7%)		14 (53.85%)		13 (54.17%)		15 (71.43%)	
	West	7 (53.85%)		0		8 (61.56%)		5 (37.71%)		4 (33.33%)	
	Southwest	5 (62.5%)		0		0		1 (12.5%)		3 (42.86%)	
Practice within the US	US	63 (71.59%)	0.027	9 (9.28%)	0.027	42 (47.19%)	0.707	41 (46.59%)	0.828	43 (56.58%)	0.993
	Non-US	16 (50%)		9 (23.68%)		17 (43.59%)		16 (44.44%)		17 (56.67%)	
Region of practice in US	Northeast	16 (84.21%)	0.515	3 (10.53%)	0.566	7 (38.89%)	0.358	10 (52.63%)	0.19	10 (71.43%)	0.012
	Southeast	20 (71.43%)		3 (6.9%)		14 (48.28%)		18 (68.58%)		16 (55.17%)	
	Mid-west	11 (73.33%)		2 (11.11%)		10 (66.67%)		6 (46.15%)		8 (80%)	
	West	11 (57.89%)		0		9 (47.37%)		6 (33.33%)		4 (23.53%)	
	Southwest	5 (71.43%)		3 (10%)		2 (25%)		1 (14.29%)		5 (83.33%)	

**Table 4:** Demographic difference among on-label and off-label BMP users.

		On-Label Use	Off-Label Use	*p* Value
BMP Use		11 (11.11%)	88 (88.89%)	-
Experience	<10	2 (18.18%)	11 (81.82%)	0.789
	>10	6 (18.75%)	26 (81.25%)	
Specialty	Orthopedics	8 (11.11%)	64 (88.89%)	0.953
	Neurosurgery	3 (11.54%)	23 (88.46%)	
Fellowship training	Yes	9 (10.59%)	76 (89.41%)	0.610
	No	2 (15.38%)	11 (84.62%)	
Practice setting	Academic	1 (5%)	19 (95%)	0.330
	Private	9 (15.79%)	48 (84.21%)	
	Both	1 (4.76%)	20 (95.24%)	
Region of fellowship in US	Northeast	1 (5%)	19 (95%)	0.222
	Southeast	2 (22.22%)	7 (77.78%)	
	Mid-west	3 (12.5%)	21 (87.5%)	
	West	1 (9.09%)	10 (90.91%)	
	Southwest	2 (40%)	3 (60%)	
Practice within the US	US	11 (15.07%)	62 (84.93%)	0.043
	Non-US	0	24 (100%)	
Region of practice in US	Northeast	2 (12.5%)	14 (87.5%)	0.609
	Southeast	2 (9.09%)	20 (90.91%)	
	Mid-west	2 (14.29%)	12 (85.71%)	
	West	4 (28.57%)	10 (71.43%)	
	Southwest	1 (14.29%)	6 (85.71%)	

**Table 5:** Respondents’ preference for BMP size for different fusion surgeries.

Size of the BMP	ALIF (80)	Multi-Level ACDF (17)	Open TLIF (60)	MIS TLIF (58)	LLIF (61)
Large (8 cc, 6 ACS, 12 mg)	5 (6.25%)	1 (5.58%)	4 (6.67%)	5 (8.62%)	4 (6.56%)
Medium (5.6 cc, 4 ACS, 8.4 mg)	11 (13.75%)	1 (5.58%)	9 (15.00%)	7 (12.07%)	8 (13.11%)
Small (2.8 cc, 2 ACS, 4.2 mg)	32 (40.00%)	2 (11.76%)	26 (43.33%)	17 (29.31%)	23 (37.70%)
X small (1.4 cc, 1 ACS, 2.10 mg)	24 (30.00%)	4 (23.53%)	10 (16.67%)	14 (24.14%)	19 (31.15%)
XX small (0.7 cc, 1/2 ACS, 1.05 mg)	8 (10.00%)	9 (52.94%)	11 (18.33%)	15 (25.86%)	7 (11.48%)

**Table 6:** Comparison of region of practice for survey correspondents in the US and estimates of surgeon distribution by region according to the AAOS.

	BMP Survey	AAOS Estimates
Southeast	33%	26%
Northeast	20%	21%
West	20%	22%
Midwest	18%	20%
Southwest	9%	11%

**Table 7:** Effect of stratification by US region on the rate of rhBMP2 use.

Variables	Survey Response	Post-Stratified Response
BMP Use	80.41%	79.57%
Case		
Single Level ALIF	71.59%	71.57%
Multi-Level ACDF	9.28%	9.23%
Open TLIF	47.19%	46.99%
Single Level MIS TLIF	46.59%	44.92%
LLIF	56.58%	57.19%

## Data Availability

Data for this study is not available.

## References

[1] Hofler R.C., Swong K., Martin B., Wemhoff M., Jones G.A. (2018). Risk of pseudoarthrosis after spinal fusion: Analysis from the healthcare cost and utilization project. World Neurosurg..

[2] Boden S.D., Zdeblick T.A., Sandhu H.S., Heim S.E. (2000). The use of rhBMP-2 in interbody fusion cages. Definitive evidence of osteoinduction in humans: A preliminary report. Spine.

[3] Ebara S., Nakayama K. (2002). Mechanism for the action of bone morphogenetic proteins and regulation of their activity. Spine.

[4] Food and Drug Administration (2007). InFUSE Bone graft/LT-CAGE Lumbar Tapered Fusion Device Approval Letter.

[5] Burkus J.K., Heim S.E., Gornet M.F., Zdeblick T.A. (2003). Is INFUSE bone graft superior to autograft bone? An integrated analysis of clinical trials using the LT-CAGE lumbar tapered fusion device. J. Spinal Disord. Tech..

[6] Ong K.L., Villarraga M.L., Lau E., Carreon L.Y., Kurtz S.M., Glassman S.D. (2010). Off-label use of bone morphogenetic proteins in the United States using administrative data. Spine.

[7] Shields L.B.E., Raque G.H., Glassman S.D., Campbell M., Vitaz T., Harpring J., Shields C.B. (2006). Adverse effects associated with high-dose recombinant human bone morphogenetic protein-2 use in anterior cervical spine fusion. Spine.

[8] Devine J.G., Dettori J.R., France J.C., Brodt E., McGuire R.A. (2012). The use of rhBMP in spine surgery: Is there a cancer risk?. Evid. Based Spine. Care J..

[9] Faundez A., Tournier C., Garcia M., Aunoble S., Le Huec J.-C. (2016). Bone morphogenetic protein use in spine surgery-complications and outcomes: A systematic review. Int. Orthop..

[10] Singh K., Ahmadinia K., Park D.K., Nandyala S.V., Marquez-Lara A., Patel A.A., Fineberg S.J. (2014). Complications of spinal fusion with utilization of bone morphogenetic protein: A systematic review of the literature. Spine.

[11] Stachniak J.B., Diebner J.D., Brunk E.S., Speed S.M. (2011). Analysis of prevertebral soft-tissue swelling and dysphagia in multilevel anterior cervical discectomy and fusion with recombinant human bone morphogenetic protein-2 in patients at risk for pseudarthrosis. J. Neurosurg. Spine.

[12] Khan T.R., Pearce K.R., McAnany S.J., Peters C.M., Gupta M.C., Zebala L.P. (2018). Comparison of transforaminal lumbar interbody fusion outcomes in patients receiving rhBMP-2 versus autograft. Spine J..

[13] Hofstetter C.P., Hofer A.S., Levi A.D. (2016). Exploratory meta-analysis on dose-related efficacy and morbidity of bone morphogenetic protein in spinal arthrodesis surgery. J. Neurosurg. Spine.

[14] Beschloss A.M., DiCindio C.M., Lombardi J.S., Shillingford J.N., Laratta J.L., Holderread B., Louie P., Pugely A.J., Sardar Z., Khalsa A.S. (2022). The Rise and Fall of Bone Morphogenetic Protein 2 Throughout the United States. Clin. Spine Surg..

[15] Cahill K.S., Chi J.H., Day A., Claus E.B. (2009). Prevalence, complications, and hospital charges associated with use of bone-morphogenetic proteins in spinal fusion procedures. JAMA.

[16] Virk S.S., Phillips F.M., Khan S.N. (2018). The influence of geography, time, and payer type on the utilization of bone morphogenetic protein (BMP) between 2005 and 2015. Clin. Spine Surg..

[17] Martin B.I., Lurie J.D., Tosteson A.N.A., Deyo R.A., Farrokhi F.R., Mirza S.K. (2015). Use of bone morphogenetic protein among patients undergoing fusion for degenerative diagnoses in the United States, 2002 to 2012. Spine J..

[18] Louie P.K., Hassanzadeh H., Singh K. (2014). Epidemiologic trends in the utilization, demographics, and cost of bone morphogenetic protein in spinal fusions. Curr. Rev. Musculoskelet. Med..

[19] AAOS census AAOS Orthopaedic practice in the U.S. 2018. https://www.aaos.org/quality/practice-management/aaos-orthopaedic-surgeon-census.

[20] Poeran J., Opperer M., Rasul R., Mazumdar M., Girardi F.P., Hughes A.P., Memtsoudis S.G., Vougioukas V. (2016). Change in off-label use of bone morphogenetic protein in spine surgery and associations with adverse outcome. Glob. Spine J..

[21] Burkus J.K., Transfeldt E.E., Kitchel S.H., Watkins R.G., Balderston R.A. (2002). Clinical and radiographic outcomes of anterior lumbar interbody fusion using recombinant human bone morphogenetic protein-2. Spine.

[22] Glassman S.D., Howard J., Dimar J., Sweet A., Wilson G., Carreon L. (2011). Complications with recombinant human bone morphogenic protein-2 in posterolateral spine fusion: A consecutive series of 1037 cases. Spine.

[23] Glassman S.D., Carreon L.Y., Campbell M.J., Johnson J.R., Puno R.M., Djurasovic M., Dimar J.R. (2008). The perioperative cost of Infuse bone graft in posterolateral lumbar spine fusion. Spine J..

[24] Smucker J.D., Rhee J.M., Singh K., Yoon S.T., Heller J.G. (2006). Increased swelling complications associated with off-label usage of rhBMP-2 in the anterior cervical spine. Spine.

[25] Crawford C.H., Carreon L.Y., McGinnis M.D., Campbell M.J., Glassman S.D. (2009). Perioperative complications of recombinant human bone morphogenetic protein-2 on an absorbable collagen sponge versus iliac crest bone graft for posterior cervical arthrodesis. Spine.

[26] Chen N.F., Smith Z.A., Stiner E., Armin S., Sheikh H., Khoo L.T. (2010). Symptomatic ectopic bone formation after off-label use of recombinant human bone morphogenetic protein-2 in transforaminal lumbar interbody fusion: Report of 4 cases. J. Neurosurg. Spine.

[27] Wong D.A., Kumar A., Jatana S., Ghiselli G., Wong K. (2008). Neurologic impairment from ectopic bone in the lumbar canal: A potential complication of off-label PLIF/TLIF use of bone morphogenetic protein-2 (BMP-2). Spine J..

